# A Rare Case of rTMS Induced Schizophrenia Symptom Switch

**DOI:** 10.1192/bjo.2024.690

**Published:** 2024-08-01

**Authors:** Aayushi Sobhani, Sachin Pursnani, Anureet kaur Chandi, Akansha Bhardwaj, Nand Kumar

**Affiliations:** All india Institute of Medical Sciences, New Delhi, India

## Abstract

**Aims:**

- This case study investigates a rare occurrence of symptom transition in a chronic schizophrenia patient following high-frequency repetitive transcranial magnetic stimulation (rTMS), aiming to understand the unexpected shift from predominantly negative to positive symptoms.
- rTMS, known for inducing changes in neuronal activity based on Faraday's law, is believed to enhance cortical excitability through high-frequency stimulation.
- Schizophrenia, a severe and chronic mental disorder, presents with both positive (e.g., delusions, hallucinations) and negative symptoms (e.g., apathy). Current treatments, predominantly antipsychotic drugs, often show limited efficacy, especially for negative symptoms. Non-invasive neuromodulation techniques like rTMS are emerging as potential interventions.

**Methods:**

This case involves a 27-year-old banking executive with a 30 months illness duration primarily marked by negative symptoms over the past 3 months. Despite various antipsychotics, there was no improvement, leading to the initiation of high-frequency rTMS on the left dorsolateral prefrontal cortex (DLPFC) as an adjunct strategy for persistent negative symptoms. Surprisingly, after the 5th rTMS session, positive symptoms like delusions and hallucinations emerged. Serial assessments demonstrated a decrease in negative symptom domain scores on PANSS but an increase in positive symptom domain scores on PANSS.

**Results:**

- Results suggest that 5 Hz rTMS over the left DLPFC may have contributed to the transition to positive symptoms. The discussion explores limited literature on rTMS-induced positive symptoms, with case reports dating back to 2004 indicating the possibility of such induction. Studies propose a link between higher pulse frequency, motor threshold intensity, left prefrontal cortex stimulation, and longer trial durations with the exacerbation of positive symptoms, possibly linked to dopamine changes in specific brain tracts.
- Recent trials indicate potential improvement in positive symptoms, such as excitement, with low frequency rTMS of the temporo parietal area. However, the efficacy of rTMS varies with the stimulation site, with left prefrontal rTMS showing promise for negative symptoms and left temporo-parietal junction stimulation possibly reducing auditory hallucinations.

**Conclusion:**

This case report suggests that a subset of schizophrenia patients may experience a transient exacerbation of positive symptoms following rTMS. This underscores the need for increased awareness of potential side effects, serving as an exploratory study that calls for future research to refine these findings for a clearer understanding of rTMS-induced symptom switches in schizophrenia.

